# Optimizing acidizing design and effectiveness assessment with machine learning for predicting post-acidizing permeability

**DOI:** 10.1038/s41598-023-39156-9

**Published:** 2023-07-22

**Authors:** Matin Dargi, Ehsan Khamehchi, Javad Mahdavi Kalatehno

**Affiliations:** Department of Petroleum Engineering, Amirkabir University of Technology, Tehran, Iran

**Keywords:** Energy science and technology, Engineering

## Abstract

Formation damage poses a widespread challenge in the oil and gas industry, leading to diminished permeability, flow rates, and overall well productivity. Acidizing is a commonly employed technique aimed at mitigating damage and enhancing permeability. In this study, to predict the permeability after acidizing in oil and gas reservoirs, three machine learning models, namely artificial neural networks, random forest, and XGBoost, along with genetic programming were used to estimate permeability changes after acidizing. These models are utilized to estimate permeability changes following acidizing operations. Training of the models involved a dataset comprising 218 acidizing operations conducted in diverse reservoirs across Iran. The input parameters, namely permeability, porosity, skin factor, calcite mineral fraction, acid injection rate, and injected acid volume, were optimized through the use of a genetic algorithm. Statistical and graphical analysis of the results demonstrates that genetic programming outperformed the other machine learning techniques, yielding superior performance with R square and RMSE values of 0.82 and 17.65, respectively. Nevertheless, the other models also exhibited commendable performance, surpassing an R square value of 0.73. The post-acidizing permeability data obtained from core flooding experiments conducted on carbonate and sandstone cores was utilized to validate the models. The genetic programming model demonstrates an average error of 21.1%. The evaluation of post-acidizing permeability using genetic programming, in comparison with the results obtained from the core-flood test, revealed errors of 22.95% and 32.4% for carbonate and sandstone cores, respectively. Furthermore, a comparison between the calculated post-acidizing permeability derived from the GP model and previous studies indicated errors within the range of 8.6–26.59%. The findings highlight the potential of genetic programming and machine learning algorithms in accurately predicting post-acidizing permeability, thereby aiding in acidizing design, effectiveness assessment, and ultimately enhancing oil and gas production rates.

## Introduction

Acidizing is a method commonly utilized in the petroleum industry to increase the permeability of oil reservoirs by eliminating formation damage^1^. Formation damage can arise at any stage of petroleum exploration and production procedures, resulting from the disparity between the injected and indigenous fluids and the mineral constituents of the formation. Matrix acidizing is a frequently employed well-stimulation technique that has been in use since the early 1920s^2,3^. By removing well-bore formation damage from drilling or fine solid migration in the matrix, its main objective is to restore permeability in the nearby well-bore region. The process involves injecting a treatment fluid into the formation, which can dissolve formation damage or create new pathways within a few inches to a couple of feet around the borehole^4^. Matrix acidizing is a low-cost, low-volume operation in sandstones and carbonate formations^1,2^. Damage can occur during the drilling, completion, or production of a well, and the primary objective of acidizing is to increase production by dissolving formation damage or creating new pathways. It is crucial to be aware of the main types of damage that occur in oil, gas, and water wells in order to identify the damage or plugging solids that require removal by a solvent^5–7^. Multiple factors influence the effectiveness of an acidizing operation, including the choice of acid, injection rate and pressure, and specific well properties^8–11^. As a result, a range of machine learning models may prove valuable in facilitating the optimization and prediction of these parameters^12^. Machine learning is the primary approach used in the field of artificial intelligence for conducting research and practical applications, as it can efficiently establish the correlation between extensive sets of data^13,14^. The use of machine learning has become increasingly popular in recent years for predicting the petro physical properties of reservoirs^15–17^. Machine learning has emerged as a valuable instrument in optimizing the process of acidizing operations through the prediction of diverse acid formulas and injection parameters. This technique leverages the analysis of historical data from wells and reservoirs to identify intricate correlations that may be elusive to human perception^17,18^. In the context of acidizing, machine learning can be used to predict the optimal combination of acid formulation, injection flow rate, and injection pressure for a given well^19^. Specifically, Artificial Neural Networks (ANNs) have proven to be effective in predicting the optimal acid formulation. ANNs are a class of machine learning models that can recognize complex patterns by simulating the behavior of neurons in the human brain. By training an ANN using historical acidizing data, it can identify the performance of different acid formulations based on the properties of the well and reservoir^5,20^. In the domain of acidization operations, support vector machines (SVMs) have been employed to anticipate the injection rate and pressure^21^. SVMs represent a type of machine learning model that is proficient at recognizing the optimal decision boundary between two categories of data. By training an SVM with historical data, it can acquire the capability to predict the injection rate and pressure that maximize production rates while mitigating the peril of impairing the well or reservoir^17,22,23^.

By combining machine learning techniques with petrophysical logs, Ahmadi and Chen’s study thoroughly compared various models for predicting porosity and permeability in oil reservoirs. The results indicate that incorporating hybridized machine learning methods in porosity and permeability estimations can result in more accurate and dependable static reservoir models for simulation plans^24^. Sidaoui et al. developed a machine learning model that achieved 90% accuracy in predicting PVBT and optimizing the injection rate for matrix stimulation using acid, while Kellogg utilized machine learning algorithms to enhance cost savings and performance in the acid maintenance program by screening candidates^21,23^. Erofeev et al. utilize machine learning to predict rock properties based on routine core analysis (RCA) data, with a two-hidden-layer neural network showing the best predictive performance^25^. Zolotukhin and Gayubov propose a machine learning-based method for determining reservoir permeability with good prediction accuracy. An analytical expression for fluid flow in reservoirs is also obtained using machine learning^26^. Hanzelik analyzed 888 oil industry rock samples and compared nine machine learning methods. XGBoost and ANN showed promising results in predicting rock solubility in acids. However, limitations include excluding non-sedimentary samples and improving mineral differentiation^5^. The challenges associated with limited sample size and indirect measurements in predicting carbonate formation permeability are overcome through the use of machine learning. The proposed correlations show promising results, with an average R square score surpassing 0.96^27^. Talebkeikhah et al. found SVM and DT models to be most accurate compared to traditional methods. Artificial intelligence techniques outperform traditional equations in permeability estimation^28^. Machine learning techniques (artificial neural networks) are shown to be more accurate and reliable in predicting permeability in tight carbonate rocks compared to conventional models. A proposed XGBoost model, optimized with particle swarm optimization, outperforms benchmark models and traditional methods for predicting tight sandstone reservoir permeability, showcasing superior performance. These findings highlight the potential of machine learning for improved permeability prediction in geoscience applications^29,30^. Mathematical models for sandstone acidizing were developed in the 1970s, but predicting the outcome of the process remains difficult due to the complexity of porous media and reactions. Gumrah et al. describe a computer model that uses a genetic algorithm to optimize Damkohler and acid capacity numbers for predicting the permeability alteration of an acidization process^31–33^. Alkathim et al. investigated the impact of rock, acid, and reaction properties on pore volume to breakthrough during calcite matrix acidizing, finding optimal injection rates^34^, while Kurniawan proposed a machine learning and regression analysis model to enhance success rates and net oil gain in hydraulic fractured sandstone formations, improving candidate selection^35^. Additionally, Abdollah Hatamizadeh and Behnam Sedaee optimized acidizing processes in carbonate reservoirs using neural networks, meta-learning algorithms, and genetic algorithms, achieving high simulation accuracy and minimizing acid consumption while enhancing permeability improvement^17^. Table 1 presents a comprehensive summary of the relevant literature pertaining to the research being conducted. This table offers a concise overview of the key studies, their inputs, model types, results, and accuracy, thereby providing valuable insights into the existing body of knowledge in the field.

**Table 1:** Literature Summary Table.

Authors	Inputs	Model types	Results	Accuracy
Ahmadi and Chen^24^	Sonic Transit Time (DT), Density Tool Reading (NPHI), Bulk Density (RHOB), PHIT (total porosity)	artificial neural network, genetic algorithm, fuzzy decision tree, the imperialist competitive algorithm (ICA), particle swarm optimization (PSO), and a hybrid of those ones	Prediction of permeability and porosity	R squared values from 0.42 to 0.99 for different models
Andrei Erofeev1 et al.^25^	Salts concentration , Formation top depth, Formation bottom depth, Porosity before desalination, Absolute permeability before desalination, Sample depth, Sample density, Average grain size, Color, Depth horizon	The models used include linear regression (with L1 and L2 regularization), decision tree, random forest, gradient boosting, neural network, and support vector machine	The best predictive ability and generalizability for all three rock characteristics (alteration of porosity, alteration of permeability, and salt concentration)	R squared values from 0.014 to 0.85 for different models
Zolotukhin and Gayubov^26^	porosity , length of sample, pressure difference , flow rate	artificial neural networks (ANN)	Prediction of permeability	R squared of 0.92
Mohsen Talebkeikhah et al.^28^	Depth, Computed gamma-ray log (CGR), Spectral gamma-ray log (SGR), Neutron porosity log (NPHI), and density log (RHOB)	Multi-Layer Perceptron Neural Network (MLP), Radial Basis Function Neural Network (RBF), Support Vector Regression (SVR), decision tree (DT), and random forest (RF)	SVM and DT models outperformed empirical equations for permeability prediction, with SVM having higher accuracy and DT having better accuracy	R squared values from 0.89 to 0.97 for different models
Abdollah Hatamizadeh and Behnam Sedaee^17^	porosity, permeability and its distribution, core dimensions, fluid saturation, oil viscosity, oil density, oil compressibility, injection rate, temperature, pressure, acid concentration, acid molecular diffusion, and reaction rate	machine learning (ML) methods, artificial neural networks (ANN), deep learning models, and meta-learning algorithms	Prediction of Pore volume to breakthrough	Models achieved accuracy ranging from 83 to 90%, with a combination of neural networks and deep learning
Candra Kurniawan et al.^35^	permeability, porosity, net formation, carbonate fraction, shale fraction, reservoir pressure, fracturing length, fracturing width, acid volume, and more	Supervised machine learning models: Neural network, logistic regression, tree, and random forest. Multivariate analysis models: Principal component regression (PCR) and partial least square regression (PLS R)	Prediction of the Successfulness of Matrix Acidizing in Hydraulic Fractured Sandstone Formation	Random forest model achieved 0.73 precision in training and 61% in validation, while PCR and PLS R models achieved 0.22 and 0.35 R square values
Murtadha Alkathim et al.^34^	Rock properties (including porosity and core length) Acid properties (including concentration and type) Reaction kinetics properties (including reaction rate constant) Acid injection rates	Two-scale continuum simulation model Machine learning techniques: Artificial neural network (ANN), fuzzy logic (FL), and support vector machine (SVM)	Estimation of the pore volume to breakthrough for carbonate acidizing	The ANN model achieved an estimation error of 11.27%, indicating its accuracy in predicting PVBT

The main goal of this study is to develop and evaluate machine learning models for predicting post-acidizing permeability, which is a crucial factor for the design and optimization of acidizing operations in oil and gas reservoirs. By using these models, engineers can gain a comprehensive understanding of the potential outcomes of acidizing before the actual operation and make informed decisions based on the projected results. The study primarily focuses on analyzing sandstone and carbonate formations. It is worth noting that the dataset available for carbonate reservoirs is larger compared to that of sandstone reservoirs. As a result, the model's accuracy is relatively higher when applied to carbonate formations, as supported by the findings of the study. This study employs operational parameters that are more accessible and relevant for predicting permeability changes than the traditional parameters used in previous studies. Genetic algorithms identify these parameters. In this study, to predict the permeability after acidizing in oil and gas reservoirs, three machine learning models, namely artificial neural networks, random forest, and XGBoost, along with genetic programming, were used to estimate permeability changes after acidizing and The post-acidizing permeability data obtained from core flooding experiments conducted on carbonate and sandstone cores was utilized to validate the genetic programming model.

## Materials

### Rock samples

The core-flood testing was conducted on two samples of carbonate and sandstone (real samples), as shown in Table 2. Before the operation, the core samples were washed using a Soxhlet apparatus to extract hydrocarbons from the solid material. The apparatus was heated to 160 °C and then lowered to 80 °C to optimize the extraction process. A solvent mixture of toluene and methanol was used to dissolve and remove hydrocarbons from the cores. The washing process lasted for two days to ensure complete cleaning of the cores. After washing, X-ray diffraction (XRD) analysis was performed on the dry rock specimens. The results of the XRD analysis are presented in Table 3.

**Table 2:** Physical characteristics of cores.

Core type	Length (mm)	Diameter (mm)
Carbonate	50	38
Sandstone	95	38

**Table 3:** XRD results.

Core type	Calcite	Quartz	Dolomite	Illite	Smectite	Mix layer
Carbonate (%)	51	29	10	4	4	2
Sandstone (%)	18	69	4	0	0	9

### Formation water

For the purpose of analyzing the chemical properties of formation water, a 1000 ml sample was prepared in accordance with the composition of actual formation water obtained from an HP/HT reservoir located in southern Iran. The sample was created by dissolving artificial compounds, as listed in Table 4, into 1000 ml of water and subsequently filtering it through a 0.4 μm filter paper. Also, The salinity of the formation water was measured to be 221,421.15 parts per million (ppm), indicating the concentration of dissolved salts in the water. Additionally, the pH of the formation water was determined to be 5.7, providing insight into the acidity or alkalinity of the water^36^.

**Table 4:** Artificial formation water compounds^36^.

Analysis	Na_2_SO_4_	NaHCO_3_	NaCl	MgCl_2_·6H_2_O	KCl	SrCl_2_	CaCl_2_·2H_2_O
concentration (g/l)	4.14	0.8812	59.53	12.547	3.43	0.29295	140.6

### Acid

To achieve a significant increase in production, the mineralogy of the formation should guide the selection of acid type for acidizing operations. In this article, the primary acids for the coreflood test are 12% HCl + 3% HF for sandstone cores and 15% HCl for carbonate cores. The selection process was based on the analysis of XRD results to ensure compatibility with the mineralogical composition of the core samples, in conjunction with the utilization of a machine learning algorithm. The inclusion of appropriate additives is also crucial for successful acidizing operations, and thus, additives such as corrosion inhibitors, iron control, and surface tension reducers were incorporated into the acid solution.

## Methodology

**Figure 1:**
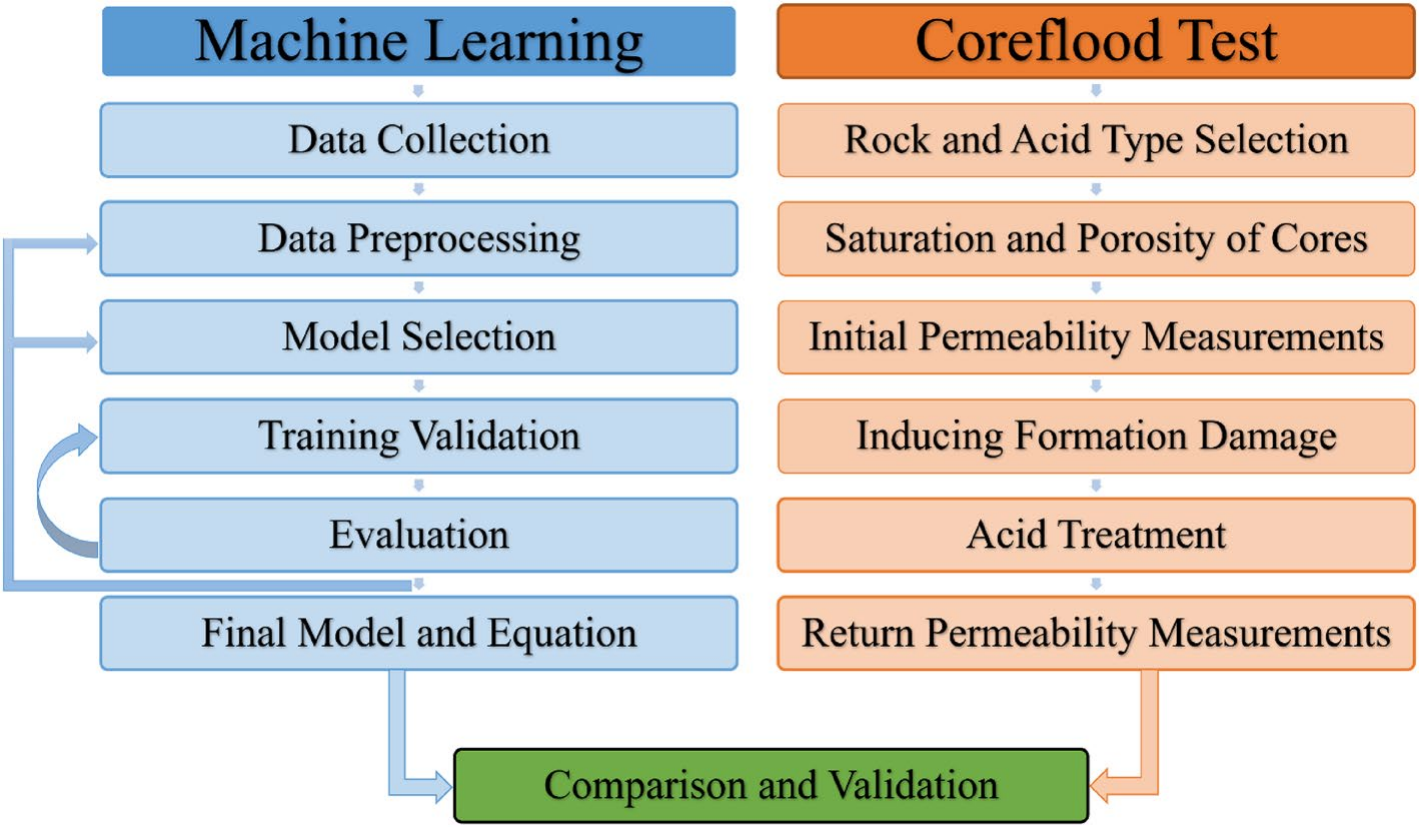
Workflow chart.

This section provides a comprehensive overview of the experimental procedures and computational techniques employed in the study. In computational techniques, genetic programming and three machine learning methods, including artificial neural networks, XGBoost, and Random Forest, were employed to develop appropriate models for predicting post-acidizing permeability using operational parameters that are new and unconventional. The performance of these models was evaluated, and the equation derived by genetic programming were compared with laboratory measurements. In the laboratory section, a validation of the results obtained from the genetic programming was conducted through the execution of two core flood tests on carbonate and sandstone cores. These tests involved the measurement of permeability before and after acidizing. Core flood tests are specialized laboratory experiments that replicate reservoir conditions, enabling the observation of the impact of acidizing on core samples. Figure 1 presents a workflow chart that facilitates a comprehensive comprehension of the concepts and processes discussed in this article.

### Computational techniques

#### Data preparing

It is a widely acknowledged fact that data preparation constitutes a crucial step in the machine learning process, as the quality of the data can significantly impact the performance of the model^37,38^. Thus, prior to feeding data into a machine learning algorithm, data cleaning and preprocessing procedures are performed to ensure optimal data quality^39^. Data cleaning encompasses the identification and handling of missing values, outliers, and irrelevant or redundant features^28,37^. Preprocessing procedures involve transforming the data into a format that the machine learning algorithm can comprehend, which may include scaling or normalizing the data to ensure that all features are on a similar scale^38^. Data normalization is a technique that involves transforming the values of a variable or feature into a new range, commonly between 0 and 1 or − 1 and 1. By scaling down the features, we ensure that they are on a standardized scale, which eliminates variations in magnitude. This standardization enables a fair comparison and combination of variables, as they are now on a common scale, facilitating accurate analysis and modeling^40^.The normalization process is performed by subtracting the minimum value of each index from its actual value, then dividing the result by the range (maximum value minus minimum value) of that index. Normalizing data allows for easy comparison of indicators with different units or magnitudes and also helps to speed up the training process^37,40^.

**Figure 2:**
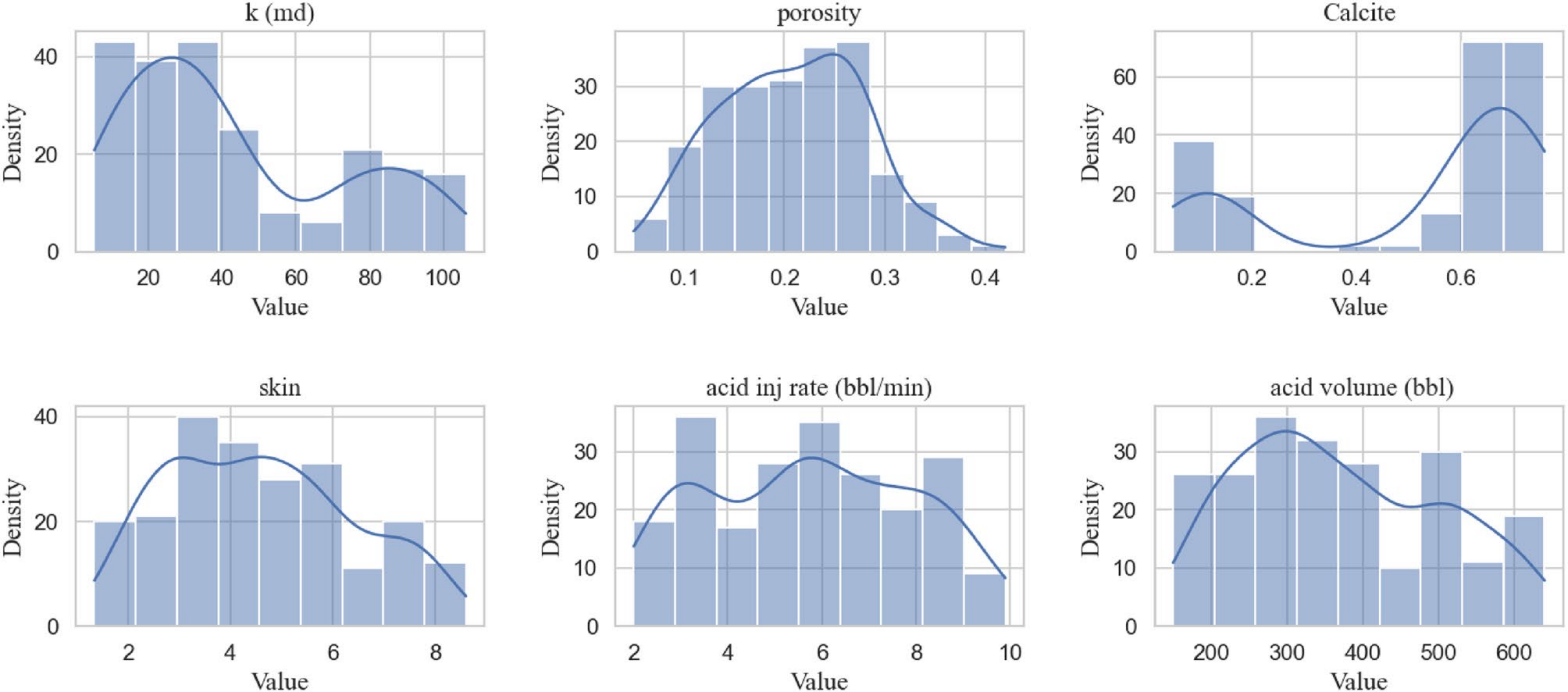
Input parameters distribution of 218 samples from Iran.

To develop machine learning models for this study, a total of 218 acidizing data samples were collected from various reservoirs located in Iran. The input variables used for the machine learning model included parameters such as initial permeability, porosity, skin factor, the fraction of calcite mineral, acid injection rate, and injected acid volume. Figure 2 presents the distribution plots for each of these parameters among the available samples. By utilizing initial permeability and skin damage as input parameters, we aimed to assess the effectiveness of acid treatment in improving permeability. While common models exist to calculate permeability when the skin factor is known, our study focuses on predicting the changes in permeability after acid treatment, taking into account the initial permeability and the impact of skin damage.

To address the presence of multiple minerals with small proportions, the decision was made to concentrate on the two primary minerals found in carbonate and sandstone formations, specifically calcite and quartz as input features. Subsequently, the quartz percentage parameter was eliminated through the use of a genetic algorithm. This choice aimed to mitigate potential adverse effects that could arise from increasing the number of input features. By restricting the number of features, the intention was to avoid issues such as overfitting, heightened computational complexity, and the curse of dimensionality. Also according to there are only two types of acid in the used data For acidizing reservoirs, these data use 15% HCl, and for acidizing sandstones, they use 12% HCl and 3% HF. Since the calcite content of the carbonate data is greater than 50% and the calcite content of the sandstone data is less than 50%, models can distinguish the type of rock and acid based on the calcite content.

The maximum permeability distribution was found to be associated with permeabilities less than 40 mD, which is consistent with the predominance of carbonate reservoirs compared to sandstone reservoirs. Moreover, Table 5 provides statistical characteristics of the data, aiding in further analysis and interpretation.

**Table 5:** The statistical details of data.

Feature	Minimum	Maximum	Average	Standard deviation	Median
k (mD)	5.3	106.1	43.27	29.12	34
Porosity (fraction)	0.05	0.40	0.21	0.07	0.21
Calcite (fraction)	0.05	0.76	0.52	0.25	0.64
Skin	1.34	8.6	4.6	1.8	4.49
Acid injection rate (bbl/min)	2	9.9	5.74	2.21	5.76
Acid volume (bbl)	150	640	371.12	130.12	350
Target variable (mD)	8.97	194.9	86.56	43.57	85.15
Target skin factor	− 1.84	− 0.01	− 1.10	0.47	− 1.35

##### Genetic algorithms to optimize dataset

**Figure 3:**
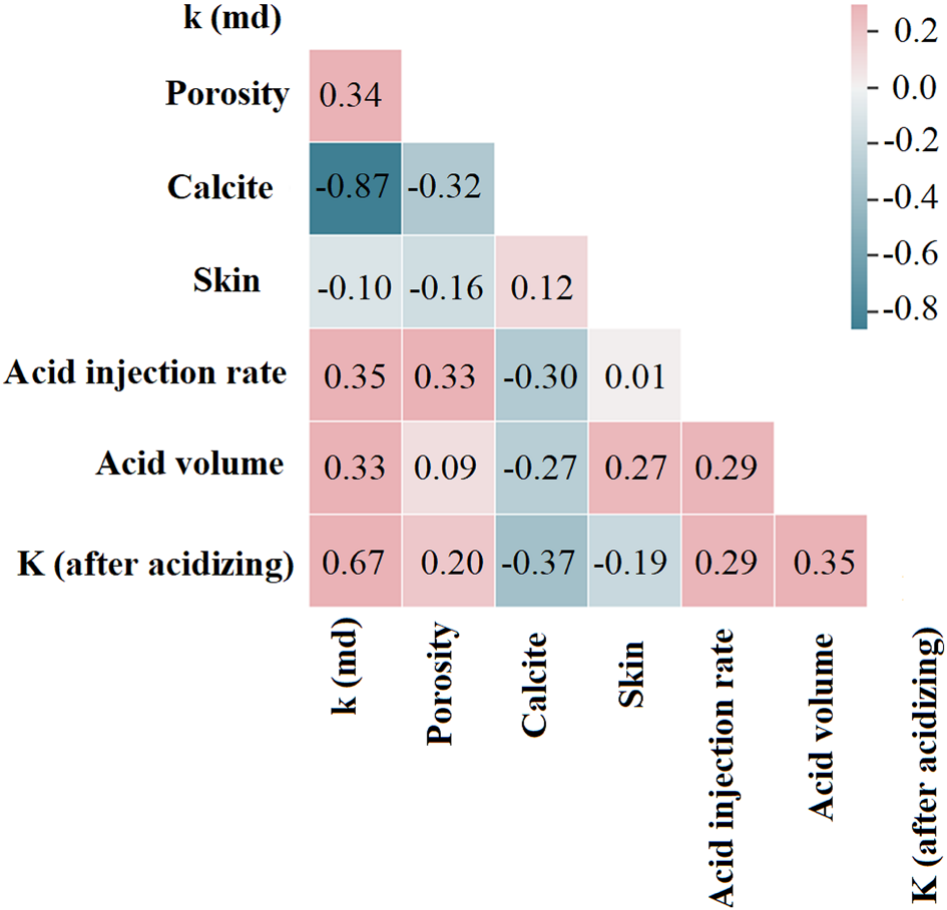
The scatter plots depicting the relationship between the selected input variables and permeability reveal the corresponding regression coefficients.

Optimizing a dataset with a genetic algorithm involves finding the best input features for a machine learning algorithm by mimicking natural selection. This involves evaluating all possible subsets of features and selecting the most promising ones for further evaluation. By doing so, we can improve the accuracy and efficiency of the machine learning model while also gaining insights into the relationships between variables in the data. Despite the challenges, optimizing datasets with genetic algorithms has shown promise in engineering and other fields. As machine learning becomes more important, using genetic algorithms for dataset optimization is likely to become more common and valuable^41,42^. The initial dataset comprised nine distinct features, which were subsequently reduced to six through the use of a genetic algorithm. The algorithm identified three parameters-the fraction of quartz, layer thickness, and formation temperature—as having negligible effects on determining the permeability value post-acidizing, leading to their exclusion from the final feature set. The process of feature reduction was found to have a considerable impact on the accuracy of the machine learning models employed. This study employed a training–testing split approach, in which 80% of the available data was randomly assigned to the training set while the remaining 20% was allocated to the testing dataset. This methodology ensures that the model is trained on a sufficient amount of data to learn patterns and trends while also being evaluated on a separate set of data to assess its generalizability and performance on new, unseen data. The split was performed randomly to ensure that the training and testing datasets are representative of the overall data distribution and to prevent any bias in the model. Notably, Fig. 3 portrays all potential associations between the chosen variables and permeability. As depicted in the figure, the regression coefficient value of the Calcite fraction and skin with respect to permeability is negative, whereas for other inputs, it shows a positive correlation.

for calcite, the negative values indicate that increasing the calcite content will reduce the target permeability (− 0.37) and acid volume (− 0.27). increasing the fraction of calcite in the rock enhances the contact between the acid and calcite. However, it is not necessary to dissolve all of the calcite, as a smaller volume of acid can effectively dissolve a certain percentage of calcite, leading to increased permeability and the formation of a wormhole. Therefore, the negative relationship between calcite content and target permeability, and acid volume can be attributed to this phenomenon. Furthermore, these relationships have been derived from the available data. Based on the data analysis, it has been observed that in carbonate reservoirs, which naturally contain higher amounts of calcite, a lower volume of acid injection has resulted in better outcomes compared to sandstones.

#### Machine learning

Machine learning has been extensively used in permeability prediction due to its ability to analyze and learn from vast amounts of data. Machine learning algorithms can identify complex patterns and correlations between input and output variables that may not be immediate. Models can be trained on large datasets, including both physical experiments and simulated data and have also been used to identify key factors that control increased permeability after acidizing, such as mineralogy, porosity, and other parameters, and their interactions. These insights can help to better understand the mechanisms controlling permeability and to design more effective strategies for enhancing or mitigating permeability in subsurface reservoirs^25,27,43^. this study utilizes genetic programming and machine learning models such as artificial neural networks, XGBoost, and random forest. These models were selected based on their proven reliability, accuracy in prediction tasks, and unique characteristics. artificial neural networks are well-suited for modeling complex relationships and capturing non-linear patterns in data, while genetic programming uses natural evolution to discover mathematical equations representing input–output relationships. XGBoost enhances performance and reduces overfitting, whereas random forest combines decision trees for robust predictions. Overall, these models were chosen due to their capabilities in handling the complexities of acidizing and their track record of accurate predictions^17,24,25,28,30,34,35,44^.

##### Artificial neural network (ANN)

**Figure 4:**
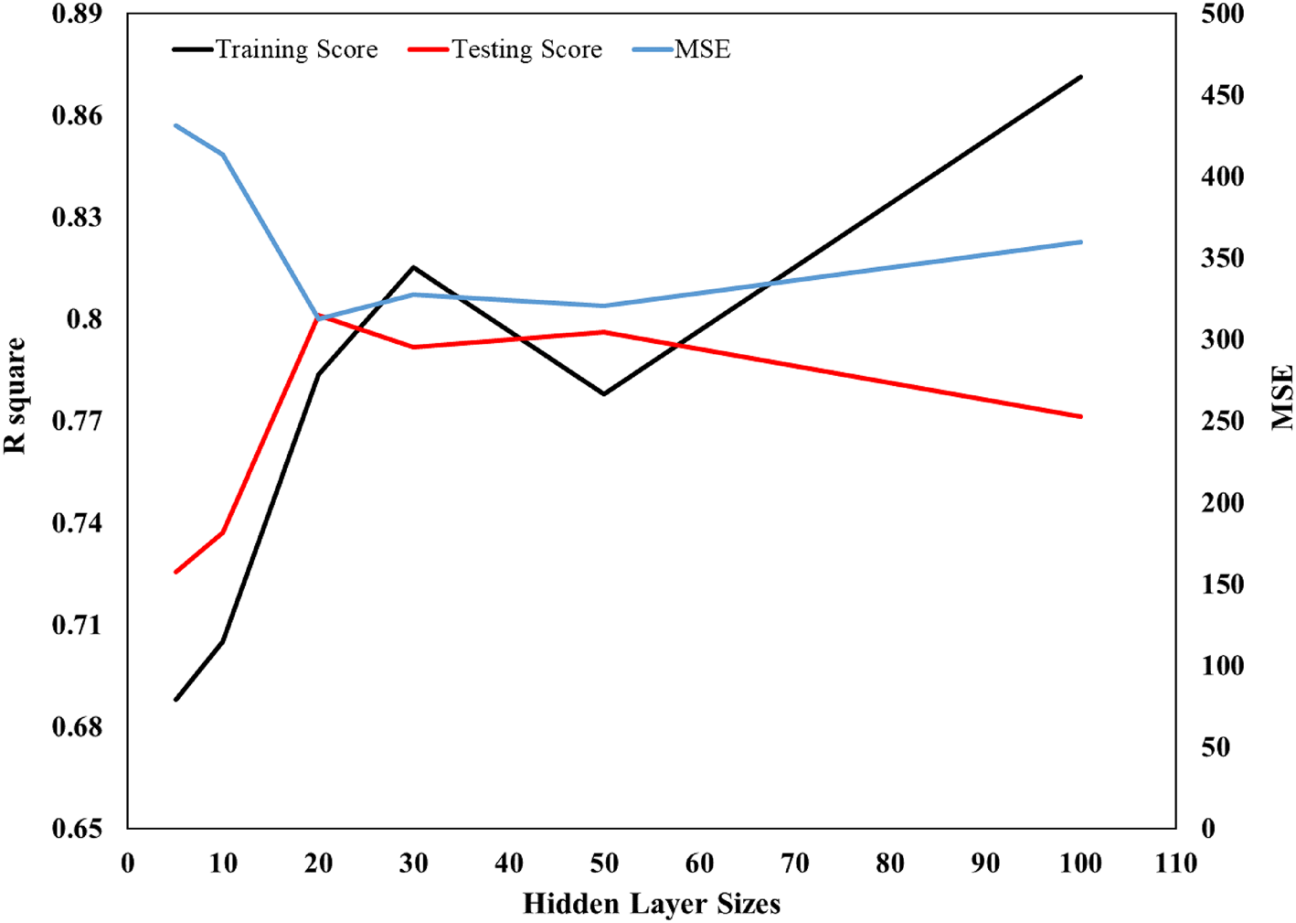
Effect of hidden layer sizes on MLP performance.

In summary, Artificial Neural Networks (ANNs) are computational models that mimic the functionality of the human brain, enabling the establishment of correlations between input and output variables in a system. To utilize ANNs for predicting permeability, the model must first undergo a training phase where the network's internal parameters are adjusted to optimize its output by minimizing the difference (error) between its predictions and the reference data. In this particular study, a set of six input parameters was employed, and the hidden layer(s) served to connect the input and output layers in the model. The complexity of the neural network model is determined by the number of neurons and hidden layers it possesses. The MLPRegressor method provided by the Scikit-learn library is a powerful implementation of ANNs for regression tasks. The method works by initializing a network with random weights and biases for the input, hidden, and output layers. The user can specify the number of hidden layers, the number of neurons in each hidden layer, the activation function, and other pertinent parameters. During the training phase, the method uses a backpropagation algorithm to update the weights and biases of the network based on the discrepancy between the predicted permeability values and the actual permeability values in the training data^24,45–48^. To achieve the best model, the R square score was plotted against the number of neurons, as shown in Fig. 4. Increasing the number of neurons improves the performance of the model during the training phase. However, this may lead to overfitting, which is evident by a significant decrease in accuracy during the testing phase. According to the figure, using a neural network model with two hidden layers and 20 neurons in each layer provides the best performance. Table 7 presents a detailed listing of the hyperparameters utilized in the selected model. Furthermore, to attain an ANN model with the utmost accuracy, an experimental design was conducted to perform a sensitivity analysis on hyperparameters. In this regard, over 100 cases were investigated, and a comprehensive summary of the sensitivity analysis can be found in Table 6.

**Table 6:** Summarization of sensitivity analysis for the ANN model.

Case	Solver (training function)	Activation	Hidden layer sizes	Number of neurons	Alpha	R-square
1	Adam	Relu	2	20	0.001	0.801
2	Adam	Relu	1	20	0.001	0.63
3	Adam	Relu	2	20	0.006	0.799
4	Adam	Relu	2	40	0.001	0.778
5	Adam	Relu	4	20	0.001	0.764
6	Adam	Identity	2	20	0.001	0.745
8	Adam	Logistic	2	20	0.001	0.37
9	Adam	tanh	2	20	0.001	0.21
10	Sgd	tanh	2	20	0.001	0.31
11	Sgd	Relu	2	20	0.001	Out of range
12	Sgd	Identity	2	20	0.001	Out of range
13	Lbfgs	Relu	2	20	0.001	0.19
14	Lbfgs	Identity	2	20	0.001	0.749
15	Lbfgs	Logistic	2	20	0.005	0.725
16	Lbfgs	Logistic	2	30	0.001	0.59
17	Lbfgs	Logistic	3	30	0.001	0.52

##### Extreme gradient boosting (XGBoost)

Extreme Gradient Boosting (XGB) is a gradient boosting algorithm that employs decision trees as base learners to form a strong learner. This study utilized XGB in conjunction with Bayesian optimization to enhance its performance. XGB not only provides parallel computing but also significantly improves algorithmic accuracy, making it widely used in various industries. The gradient boosting method implemented in this study utilized the XGBoost library, which allows for regularization to be added to the model. Finally, the model was developed by combining the first estimation with all subsequent estimations using appropriate weights^45,49–51^. Table 7 provides a comprehensive inventory of the hyperparameters used in the chosen model.

**Table 7:** Hyperparameters used in the models.

Algorithms	Hyperparameter	Definition	Value
GP	Population_size	The number of individuals in the population	50,000
	Generations	The number of generations or iterations of the genetic programming loop	30
	p_crossover	The probability of performing a crossover operation during reproduction	0.7
	p_subtree_mutation, p_hoist_mutation, p_point_mutation	The probability of performing a subtree, hoist , point mutation operation during reproduction	0.1
ANN	Number of layers	–	3
	Number of neurons in 1st hidden layer	–	20
	Number of neurons in 2nd hidden layer	–	20
	activation	The activation function for the hidden layers	relu
	Solver	The optimization algorithm used for training the neural network	adam
	Alpha	The L2 regularization parameter	0.001
XGBoost	Objective	The objective function to be minimized during training	reg:squarederror
	Colsample_bytree	Subsample ratio of columns when constructing each tree	0.4
	Learning_rate	Learning rate or shrinkage rate	0.1
	n_estimators	Number of trees in the ensemble	100
	Alpha	L1 regularization term on weights	10
RF	n_estimators	The number of trees in the random forest. In the code, it is set to 100	100
	Max_features	The number of features to consider when looking for the best split	sqrt

##### Random forest (RF)

The random forest algorithm is based on building multiple decision trees independently using bootstrap resampling to prevent overfitting. Each tree is constructed using a subset of the data, and the trees are combined by averaging their predictions to obtain the final result. This algorithm, which is implemented in the Python scikit-learn library as the RandomForestRegressor() method, has the added benefit of feature ranking. Breiman initially introduced the application of random forest as a set of unpruned decision trees with sequential growth instead of a single restricted type. The bootstrap sampling method is used in RF to randomly select data with replacement, while the remaining data is used for testing. This process is repeated for all trees, resulting in improved estimation due to the differences between sets of trees^45,51,52^. Table 7 provides an exhaustive listing of the hyperparameters utilized by the selected model.

#### Genetic programming (GP)

Genetic programming (GP) is a computational method that employs a population of computer programs represented as tree structures to discover mathematical expressions fitting a given dataset^53^. Through evolutionary operators like crossover, mutation, and selection, GP modifies program encodings to generate improved offspring and optimize solutions^54,55^. It provides insights into the input–output relationship, enhancing system performance evaluation. GP evolves populations using principles similar to genetic algorithms, where individuals' fitness is assessed based on their performance in the environment. The creation of each generation involves selecting fit individuals and breeding them through genetic operators^56^. The process continues until a termination criterion, such as a maximum generation limit or allowable error, is met. The best program in the final population is considered the result of the GP process^57^.

**Figure 5:**
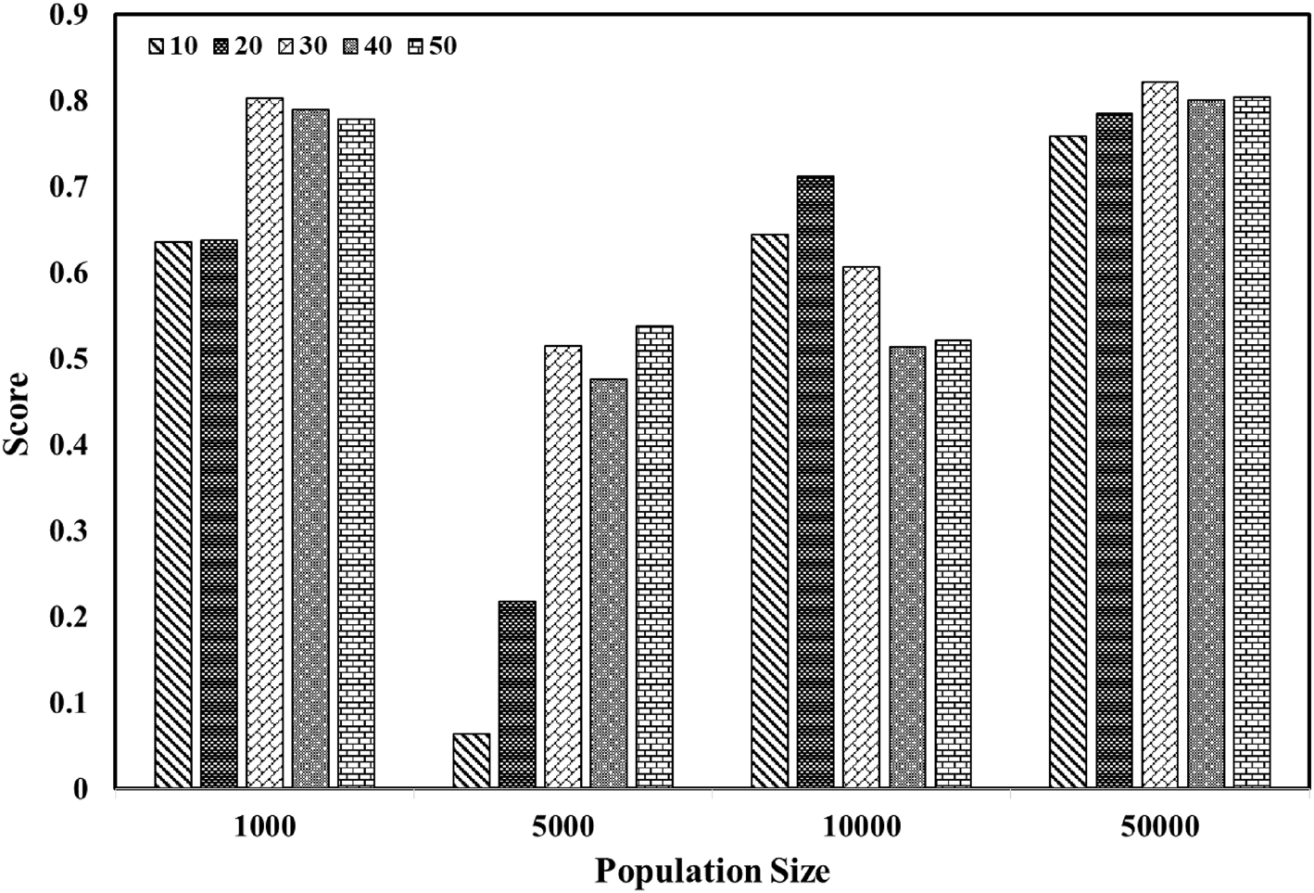
Effect of population size & number of generations on GP performance.

In this study, the optimal initial population size and generation number, which provide the highest accuracy for the model, were determined using Fig. 5. As evident from the figure, a model with an initial population size of 50,000 and a generation number of 30 demonstrated the best performance. Therefore, increasing the initial population size and generation number does not necessarily lead to an increase in accuracy. The hyperparameters utilized by the selected model are exhaustively listed in Table 7.

### Core-flood experiment

Formation damage is a prevalent operational and economic concern that can lead to a decrease in permeability within hydrocarbon formations due to incompatible processes. This issue can arise at various stages of oil and gas production in underground reservoirs^36^. To mitigate formation damage, acidizing is commonly employed. The process involves the use of acids that react with the formation, thereby opening up the pore throats and removing damage, which ultimately enhances permeability. In carbonate formations, acid can completely eliminate damage and even dissolve some of the rock beyond its undamaged state, leading to further increases in permeability. However, in sandstone formations, selective acidizing can only ameliorate formation damage. This study aimed to assess the impact of formation damage on permeability and identify potential solutions through a core-flood experiment. The experiment involved the use of two cores made of carbonate and sandstone, which were saturated with formation water prior to measuring their main parameters and initial permeability based on Darcy’s law. Subsequently, the Vinci FDS 350 device was utilized to artificially induce formation damage in the core, and thereafter, chosen acid solutions were injected into the cores to ameliorate the damage. The core-flood experiments were conducted under a pressure differential of 125 psi and a temperature of 200 degrees Fahrenheit. Following the experiment, the return permeability of the cores was measured using a similar method of formation water penetration as that used during the initial permeability measurement.

## Results and discussion

### Machine learning

**Figure 6:**
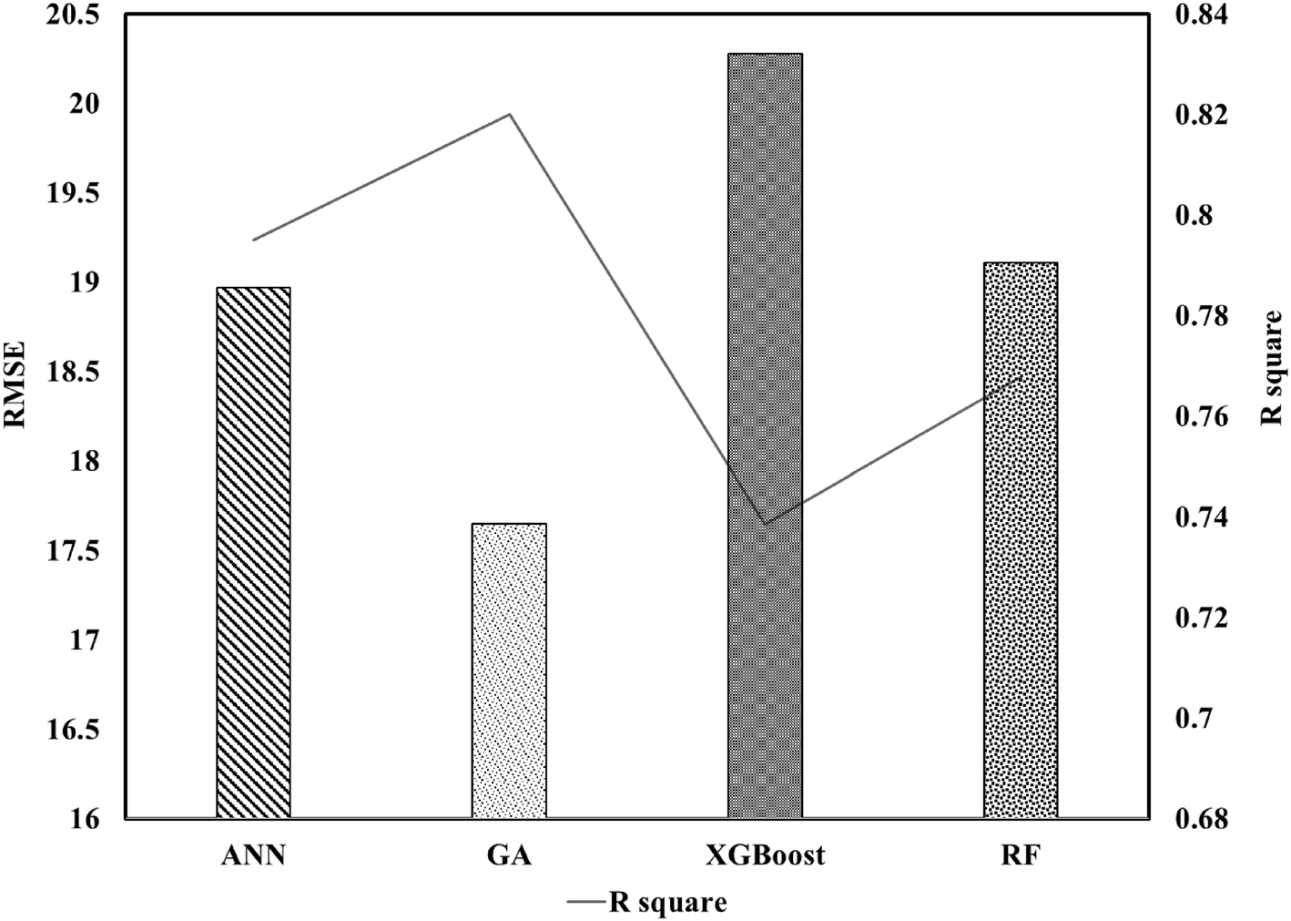
Permeability prediction metrics for all machine learning techniques.

In this section, the performance of genetic programming and three machine learning models in predicting permeability after acidizing, which were introduced in the methodology section, are presented and compared. As shown in Fig. 6, the highest accuracy among the applied models belongs to genetic programming with an R-squared value of 0.82, and the lowest value belongs to the XGBoost algorithm with an R-squared value of 0.73. Additionally, the neural network and random forest algorithms show near performance with RMSE values of 18.97 and 19.1, respectively.

**Figure 7:**
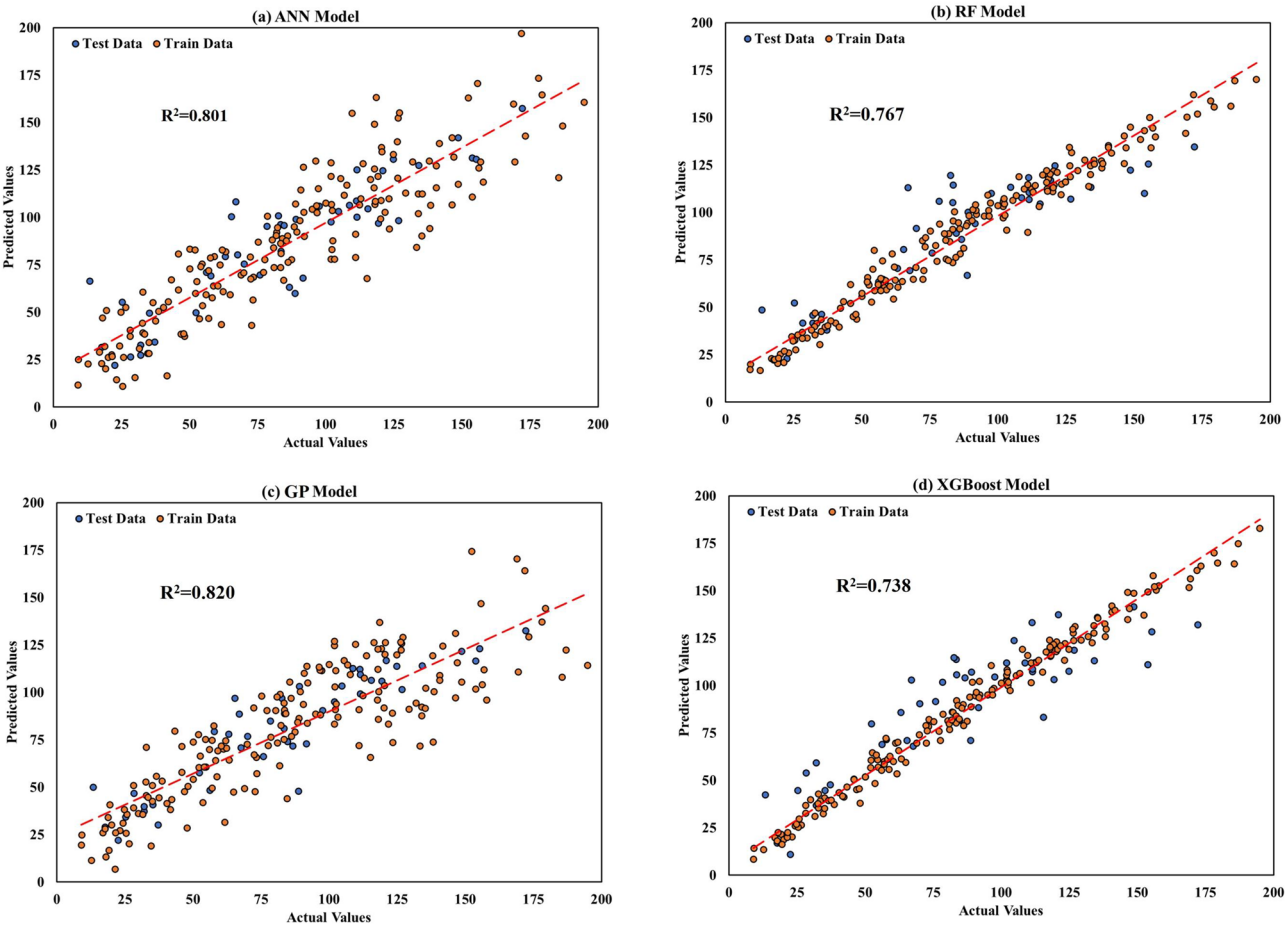
The cross plot of modeling prediction of permeability versus measured data. For (**a**) ANN, (**b**) RF, (**c**) GP and (**d**) XGBoost.

Figure 7 illustrates the plot of actual data versus predicted data in the part of the dataset where the used methods perform best, providing a visual insight into permeability prediction.

The plot shows the predicted values on the vertical axis and the measured values on the horizontal axis, along with their regression plot. The permeability values of the test data and train data have been depicted in graphical form using blue and orange markers, respectively. The plot indicates that the GP model has the best match between measured and predicted data. Many machine learning methods are considered “black boxes” because the relationship between the input parameters and the output is not easily understood. As a result, there is growing interest in explainable machine learning. One approach to enhancing model interpretability is through parameter importance analysis, which can identify the most influential input parameters on the model output. This analysis estimates the reduction in model accuracy when a particular input parameter is omitted, thereby identifying the inputs that have the greatest positive or negative impact on the output^44^.

**Figure 8:**
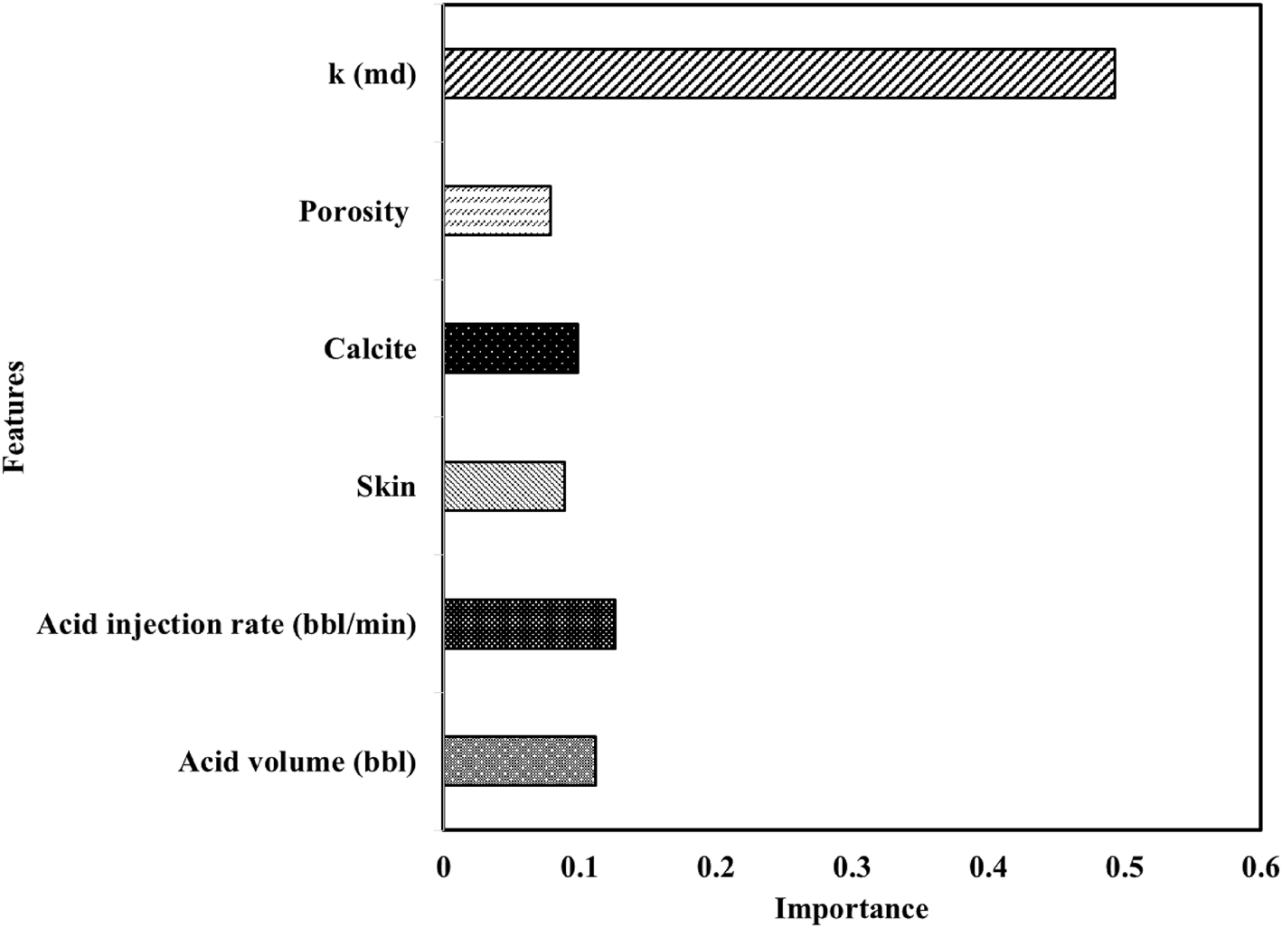
Feature importance of Random forest model.

In this study, a feature importance analysis was conducted on the model by a random forest algorithm that has an R-square value of 0.76, and the results presented in Fig. 8 showed that permeability was the most important feature, followed by acid injection rate, while porosity was found to be the least important feature. This type of analysis can help researchers better understand how the model works and identify areas for improvement.

The neural network model employed in this study consists of two hidden layers, each comprising 20 neurons. As shown in Fig. 4, The optimal performance of the model during the testing phase was observed with this configuration, where the values of R-square and RMSE were found to be 0.801 and 18.97, respectively. Figure 5 displays the model’s performance, depicting a reasonable agreement between the permeability predicted by the model and the permeability obtained from real data. Compared to other algorithms, the genetic programming utilized in this study demonstrates superior performance. A population size of 50,000 and 30 generations are employed in this model. A noteworthy characteristic of the genetic programming is the provision of a suitable equation to calculate the output parameter. In this work, Eq. (1) represents the final form of the equation presented by the model after modifications, simplification, and optimization of its coefficients.

1 $$ k = \frac{A + B + C - 0.439}{D} $$

where k_i_ is the initial permeability and x is the calcite fraction. Furthermore, the parameters A, B, and C are calculated from Eqs. (2), (3), and (4). Also, the D parameter is equal to 12.7 for ki between 5.3 mD to 60 mD and 17.07 for ki between 60 to 106 mD.

2 $$ A = 4.243xk_{i} $$

3 $$ B = x + k_{i} + 4.243 $$

4 $$ C = \left( {\frac{{k_{i} + xk_{i} + B}}{0.103}} \right) $$

The equation presented earlier can accurately calculate post-acidizing permeability using two input parameters: initial permeability and calcite frequency, with an accuracy of 82%. Despite Eq. (1) being a function of only two parameters, it was developed using genetic programming and includes all input features. Therefore, the developed equation is based on complex relationships between features and the simplification of the presented equation.

### Core-flood experiment

Within this section, the primary parameters of the core as well as the initial permeability (as per Darcy’s law) were assessed via the Vinci FDS 350 device, and the outcome of the evaluation has been documented in Table 8.

**Table 8:** Core sample properties.

Core type	Pore volume (cm^3^)	Porosity (%)	Grain density (gr/cm^3^)	Initial permeability (mD)
Carbonate	2.769	4.883	2.650	10.52
Sandstone	18.413	17.090	2.746	53.92

As shown in Table 8, two cores with different pore volumes were selected for the core-flood test. After saturating the cores with formation water and evaluating the initial parameters, condensate oil was injected into the cores to induce formation damage. Then, the secondary permeability was measured after creating formation damage, which was similar to the primary permeability. After that, acid was injected into the cores in the opposite direction of the measured permeability. Following acid injection, the return permeability was measured, which was similar to the primary permeability for both cores. The results of this experiment are reported in Table 9.

**Table 9:** Core-flood results.

Core type (1)	Acid type (2)	Initial permeability (3) (mD)	Secondary permeability after condensate injection (4) (mD)	Skin Damage	Return permeability after acid injection (5) (mD)	Skin stimulation	Reduction permeability ((3–4)/3) (%)	Increased permeability compared to initial permeability ((5–3)/5) (%)	Increased permeability compared to condensate injection ((5–4)/4) ()
Carbonate	HCl 15%	10.52	6.34	1.855	21.78	− 1.994	39.73	51.7	243.5
Sandstone	HCl 12% + HF 3%	53.92	50.025	0.269	56.12	− 0.375	7.22	3.92	12.18

The evaluation of secondary permeability in two types of plugs, sandstone and carbonate, revealed a significant reduction in permeability due to the penetration of condensate. Specifically, the reduction was calculated to be 7.22% and 39.73% for sandstone and carbonate plugs, respectively. Additionally, the extent of permeability reduction resulting from skin damage was assessed using the Hawkins equation for two core samples^58^. The findings indicate that the skin damage caused by the infiltration of condensate into the core is measured at 1.855 for carbonate cores and 0.269 for sandstone cores.The findings of this study suggest that the reduction in permeability, which is indicative of an increase in damage, was more pronounced in the carbonate reservoir than in the sandstone reservoir. This discrepancy can be attributed to the comparatively greater pore volume of the sandstone reservoir relative to that of the carbonate reservoir. Consequently, as a result of its bigger pore volume, the sandstone reservoir experienced less obstruction from oil emulsion within its pores. To mitigate this issue, it is necessary to dissolve a portion of the rock and remove the condensates from the pores through acid injection. In this study, HCl 15 wt% was utilized for the carbonate plug while HCl 12 wt% + HF 3 wt% was used for the sandstone plug. Two core-flood tests were conducted with these acids, incorporating additives such as corrosion inhibitors, corrosion inhibitor intensifiers, iron control agents, and surface tension reducers. The results indicated that injecting HCl 15 wt% and HCl 12 wt% + HF 3 wt% into core plugs resulted in an increase in permeability by 51.7% and 3.92%, respectively, compared to their initial state. Furthermore, compared to the state where formation damage occurred, there was a remarkable improvement in permeability by up to 243.5% and 12.18%, respectively. Moreover, the extent of skin stimulation, aimed at enhancing permeability following the acidizing test, was evaluated for two core samples using the Hawkins equation^58^. The results indicate that the stimulation skin values for carbonate and sandstone cores are − 1.994 and − 0.375, respectively. The findings of this study indicate that selective acids have the capacity to eliminate damage in both carbonate and sandstone reservoirs, as well as dissolve a portion of the stone. However, it was observed that the degree of stone dissolution in sandstone reservoirs was considerably lower than in carbonate reservoirs. This discrepancy can be attributed to the fact that in carbonate reservoirs acid readily reacts with calcite and enhances the porosity of the stone. Conversely, in sandstone reservoirs, due to the limited presence of calcite and the prevalence of quartz, acid is unable to dissolve a substantial amount of stone.

**Figure 9:**
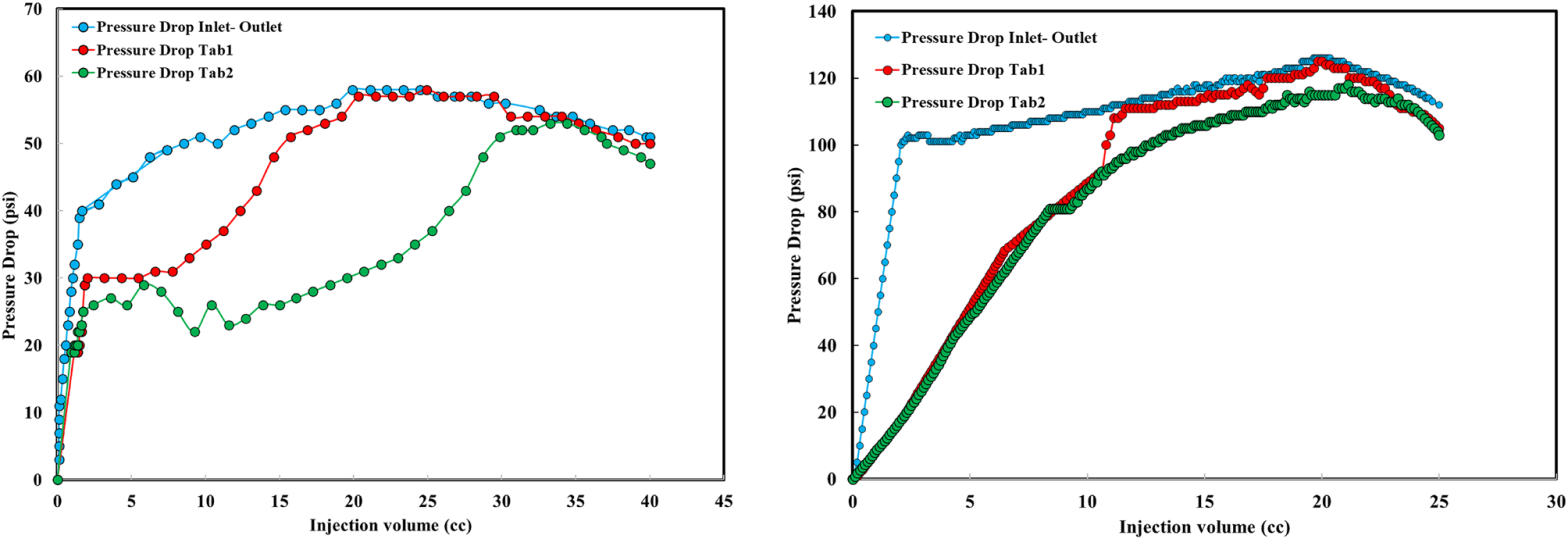
*Left:* Pressure drop versus Injection volume for sandstone core. *Right:* Pressure drop versus Injection volume for carbonate core.

In order to evaluate the outcomes, a graph was constructed to illustrate the relationship between pressure drop and injection volume. The measurements of pressure drop for both sandstone and carbonate cores during injection were recorded and depicted in Fig. 9.

Figure 9 depicts the pressure variations observed by three pressure sensors, namely Pressure Drop Inlet–Outlet, Pressure Drop Tab1, and Pressure Drop Tab 2, located on the plug holder. The initial stage of the experiment involves the fluid reaching the back of the plug (where it is considered as a well), which results in a pressure drop on both sides of the plug as recorded by Pressure Drop Inlet–Outlet sensor. Similarly, Pressure Drop Tab 1 and Pressure Drop Tab 2 also register a pressure drop. However, until the fluid reaches these two sensors, their pressure drop is comparatively lower than that of Pressure Drop Inlet–Outlet. This can be attributed to the fact that Pressure Drop Tab1 is situated closer to the start of the plug and thus experiences a quicker reduction in pressure compared to Pressure Drop Inlet–Outlet. Subsequently, as more fluid penetrates into the plug over time, Pressure Drop Tab 2's pressure drop eventually reaches that of Pressure Drop Inlet–Outlet and Pressure Drop Tab 1's pressure drop. Eventually, due to rock dissolution, all three sensors exhibit a decreasing trend in their respective curves. The significant reduction in flooding pressure following treatment confirms successful flow establishment.

### Comparison of genetic programming and laboratory results

With the application of machine learning techniques, Eq. (1) was derived. Subsequently, the outcomes of Eq. (1) were juxtaposed with those obtained from core-flood experiments, and a thorough examination of the findings was conducted. The results of this meticulous analysis are presented in Table 10.

**Table 10:** Comparison of machine learning models and laboratory results.

Core type (1)	Acid type (2)	Initial permeability (3) (mD)	Return permeability after acid injection (4) (mD)	Permeability calculated from Eq. 1 (mD)	Error (%)
Carbonate	HCl 15%	10.52	21.78	26.78	22.95
Sandstone	HCl 12% + HF 3%	53.92	56.12	74.33	32.4

Table 10 presents the results of the acidizing test carried out on two distinct core samples, namely sandstone and carbonate. The permeability values obtained after the test for these samples are recorded as 56.12 and 21.87 millidarcies, respectively. Furthermore, the calculated permeability values from Eq. (1) for these two cores are noted as 26.78 and 74.33, respectively. An analysis of the percentage of error based on the permeability values derived from the test and the calculated values from the equation indicates a discrepancy of 32.4% and 22.5% for the sandstone and carbonate cores, respectively. Compared to the machine learning model using genetic programming and the resulting equation, which had an error rate of 21.1%, the calculated error values for the difference in permeability obtained from the equation and the coreflood test were relatively acceptable and close to the expected error for the sandstone and carbonate samples. However, a larger difference was observed in the sandstone sample, which was due to the skin factor being outside the range (less than 1.34).

Table 11 presents a comprehensive comparison between the results derived from the equation obtained through genetic programming and the findings from previous studies.

**Table 11:** Comparison of machine learning models and laboratory results.

Row	Initial permeability (mD)	Return permeability after acid injection (mD)	Permeability calculated from Eq. 1 (mD)	Error (%)
1	10	18.5	20.09	8.6
2	9.8	18.11	20.11	11.05
3	46.9	93.8	118.74	26.59
4	46.9	93.8	110.36	17.66
5	46.9	93.8	105.12	12.07

In one study, dolomite rock with 10 mD permeability demonstrated an 85% increase in permeability due to hydrochloric acid penetration. When comparing the observed increase in permeability to the values predicted by the developed equation, Table 11, rows 1, revealed an error percentage of 8.6%^59^. Another investigation by Shafiq et al. focused on dolomite rock with 9.8 mD permeability, resulting in an increase to 18.11 with hydrochloric acid penetration. The observed increase was compared to predicted values, yielding an error percentage of 11.05% (Table 11, rows 2)^60^. Furthermore, a study conducted by Al-Anazi et al. (1998) explored calcitic rock permeability and discovered a twofold increase with 15% hydrochloric acid penetration. While specific information about the calcite percentage was not provided in their article, comparative analysis considered calcite percentages of 50, 60, and 76. Comparing the reported permeability increase in Al-Anazi et al.'s research to the predictions obtained from the developed equation resulted in an error percentage ranging from 12.07 to 26.56% (Table 11, rows 3–5)^61^.

## Limitations

It is important to highlight that the developed models and equation in this study are subject to certain limitations arising from the constrained training data utilized in the machine learning model. These limitations encompass:

1. *Applicability to Specific Reservoirs* The derived equation is specifically applicable to sandstone reservoirs that have undergone acidization using a combination of 12% hydrochloric acid and 3% hydrofluoric acid, as well as carbonate reservoirs treated with 15% hydrochloric acid.
2. *Permeability and Calcite Frequency Range* The models and equation are valid within a permeability range of 5.3–106 and a corresponding calcite frequency range of 0.05–0.76.
3. *Exclusion of Insignificant Minor Minerals* In order to address concerns associated with overfitting, heightened computational complexity, and the curse of dimensionality in the constructed models, minor minerals that do not significantly contribute to the rock composition have been intentionally excluded.
4. *Temperature Relationship* Given the close proximity of temperature values observed in the wells utilized for this study, no significant relationship between temperature and post-acidizing permeability was identified. Consequently, temperature was not included as one of the influential input factors for predicting permeability after acidification.
5. *Applicability Range* It should be noted that the models presented in this paper are valid only within the range of values specified in Table 5. Extrapolating the equations beyond this range may yield unreliable results.

## Conclusion

In conclusion, to predict the permeability after acidizing in oil and gas reservoirs, three machine learning models, namely artificial neural networks, random forest, and XGBoost, along with genetic programming, were used to estimate permeability changes after acidizing and The post-acidizing permeability data obtained from core flooding experiments conducted on carbonate and sandstone cores was utilized to validate the genetic programming model. Key findings of this research include:

1. Optimization of the machine learning models’ input parameters using genetic programming led to improved accuracy and performance. The number of input features was reduced to six, eliminating parameters such as quartz fraction, temperature, and layer thickness.
2. R SQUARE and RMSE values of 0.82 and 17.65, respectively, show that genetic programming outperformed the three machine learning techniques (ANN, RF, and XGBoost), demonstrating the best performance. However, the other models also exhibited relatively good performance, with R SQUARE values exceeding 0.73.
3. The genetic programming model emphasized the importance of initial permeability and calcite fraction, as reflected in the developed relationship. On the other hand, the RF model highlighted initial permeability and acid injection rate as significant features. This indicates that the importance of features may vary across different machine learning algorithms.
4. The calculated values of permeability after acidizing using the genetic programming equation showed an error of 32.4% for sandstone samples and 22.95% for carbonate samples compared to the measured values obtained from the core-flood experiment. Considering the 21.1% error of the genetic programming model itself, these differences were relatively close and deemed acceptable. Thus, the proposed equation for calculating permeability after acidizing is considered valid.
5. Further validation of the developed formulation was performed by comparing the equation with previous studies, yielding an error percentage below 26.6%. This comparative analysis provides additional confirmation of the accuracy and reliability of the developed approach.

In conclusion, the machine learning models and genetic programming offer a robust framework for predicting permeability alterations after acidizing. The findings of this study contribute to the understanding and optimization of acidizing processes in sandstone and carbonate reservoirs, paving the way for enhanced reservoir management strategies in the oil and gas industry.

## Data Availability

The datasets used and/or analyzed during the current study available from the corresponding author on reasonable request.
